# Expression scoring of a small‐nucleolar‐RNA signature identified by machine learning serves as a prognostic predictor for head and neck cancer

**DOI:** 10.1002/jcp.29462

**Published:** 2020-01-14

**Authors:** Lu Xing, Xiaoqi Zhang, Xiaoqian Zhang, Dongdong Tong

**Affiliations:** ^1^ Shandong Key Laboratory of Oral Tissue Regeneration, School of Stomatology Shandong University Jinan Shandong China; ^2^ State Key Laboratory of Oral Disease, Department of Orthodontics, West China Hospital Stomatology Sichuan University Chengdu Sichuan China; ^3^ Department of Stomatology Haiyuan College of Kunming Medical University Kunming Yunnan China; ^4^ Department of Oral and Maxillofacial Surgery, School and Hospital of Stomatology Shandong University & Shandong Key Laboratory of Oral Tissue Regeneration & Shandong Engineering Laboratory for Dental Materials and Oral Tissue Regeneration Jinan Shandong China

**Keywords:** biomarker, head and neck squamous cell carcinoma, noncoding RNA., prognosis, snoRNA, survival

## Abstract

Head and neck squamous cell carcinoma (HNSCC) is a common malignancy with high mortality and poor prognosis due to a lack of predictive markers. Increasing evidence has demonstrated small nucleolar RNAs (snoRNAs) play an important role in tumorigenesis. The aim of this study was to identify a prognostic snoRNA signature of HNSCC. Survival‐related snoRNAs were screened by Cox regression analysis (univariate, least absolute shrinkage and selection operator, and multivariate). The predictive value was validated in different subgroups. The biological functions were explored by coexpression analysis and gene set enrichment analysis (GSEA). One hundred and thirteen survival‐related snoRNAs were identified, and a five‐snoRNA signature predicted prognosis with high sensitivity and specificity. Furthermore, the signature was applicable to patients of different sexes, ages, stages, grades, and anatomic subdivisions. Coexpression analysis and GSEA revealed the five‐snoRNA are involved in regulating malignant phenotype and DNA/RNA editing. This five‐snoRNA signature is not only a promising predictor of prognosis and survival but also a potential biomarker for patient stratification management.

## Graphical abstract

Small nucleolar RNAs signature predicts the survival of head and neck squamous cell carcinoma.

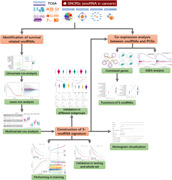

## 1 INTRODUCTION

Head and neck cancers include tumors from the oral cavity, pharynx, and throat, and 90% of the tumors belong to squamous cell carcinoma. Being the sixth most common cancer worldwide (Cai, Dodhia, & Su, 2017; Moy, Moskovitz, & Ferris, 2017), over 600,000 cases of head and neck squamous carcinoma (HNSCC) were diagnosed each year, from which more than two‐thirds of them are from developing countries (J. Moskovitz, Moy, & Ferris, 2018). Patients who were diagnosed with HNSCC are approximately 60 years old, with incidence higher in males than that in females (Polanska et al., 2014). HNSCC is responsible for about 350,000 deaths each year, indicating that its high mortality rate as well as poor prognosis a heavy burden for the whole society (Wilkie, Lau, Vlatkovic, Jones, & Boyd, 2018). At present, the primary therapeutic strategies treating HNSCC include surgical resection, radiotherapy, chemotherapy, and biotherapy (J. M. Moskovitz, Moy, Seiwert, & Ferris, 2017). However, due to a lack of efficient early diagnostic tools such as predictive markers, most of the HNSCC patients are diagnosed at late stages, losing the best opportunity for early intervention. Furthermore, poor patient management and uneven medical resources distribution can be found on HNSCC patients since very little is known about the patients’ prognosis after diagnosis. (Bunbanjerdsuk et al., 2019; Huang & O'Sullivan, 2017; Xing, Zhang, & Tong, 2019). Thus, it can be concluded that despite recent advances in therapeutic management of HNSCC, little is improved in the overall survival (OS) of HNSCC patients due to the lack of diagnostic and prognostic markers (Galot et al., 2018), which means that it is currently quite urgent to identify and apply early diagnostic as well as prognostic signature for HNSCC patients.

Over the past few years, noncoding RNAs, especially micro RNAs (miRNAs) and long noncoding RNAs (lncRNAs), have been revealed as promising biomarkers for the diagnosis and prognosis of diseases, including various cancers (He et al., 2018; G. Liu et al., 2018; Xing, Zhang, & Chen, 2019). However, another class of noncoding RNAs, small nucleolar RNAs (snoRNAs), have rarely been considered as biomarkers for cancers due to the long‐time common belief that they only perform housekeeping functions. snoRNAs are small RNAs of 60–300 nucleotides in length and are mainly found in the nucleolus (Williams & Farzaneh, 2012); they are one of the best‐characterized classes of noncoding RNAs, and their function in rRNA biogenesis has been well documented (Romano, Veneziano, Acunzo, & Croce, 2017). Growing evidence has demonstrated that snoRNAs also play an important role in the carcinogenesis of multiple tumors(Mei et al., 2012). Dong et al have indicated that the mutation or downregulation of snoRNA U50 is associated with a malignant phenotype and identified snoRNA U50 as a candidate tumor‐suppressor gene in prostate cancer (Dong et al., 2008). Another study focused on SNORA42, an H/ACA box snoRNA encoded at 1q22, whose expression is frequently increased in non‐small‐cell lung cancer (NSCLC; Testa et al., 1997), while the small interfering RNA (siRNA)‐induced downregulation of SNORA42 in NSCLC cell lines was able to induce apoptosis and reduce colony formation in vitro and was observed to inhibit tumor formation in a mouse model (Mei et al., 2012). Additionally, another study found that SNORA42 also acts as an oncogene in colorectal cancer(Okugawa et al., 2017). SNORA21 appears to have potential as a prognostic biomarker in colorectal cancer according to Yoshida et al. (2017). Recently, a systematic pan‐cancer analysis of the expression of snoRNAs in human cancer has highlighted significant roles of snoRNAs in the development and implementation of biomarkers or therapeutic targets for cancer (Gong et al., 2017). Considering the small size and stability of snoRNAs, as well as the accumulating evidence of its potential role in multiple tumors, snoRNAs are gaining increasing attention in the field of oncology and have the potential to serve as biomarkers for diagnosis, prognosis and therapeutic targets.

In our study, we were the first to perform a series of machine learning analyses to explore and construct a prognostic signature based on snoRNAs to predict the survival of HNSCC patients in 510 HNSCC patients. We identified prognosis‐related snoRNAs using univariate regression analysis. Then, we performed dimensionality reduction for significant prognosis‐related snoRNAs through the least absolute shrinkage and selection operator (LASSO) regression and multivariate regression and constructed a five‐snoRNA‐based prognostic signature. Finally, we assessed the clinical utility of this prognostic model and explored its potential functions. Our findings provide new insights into the clinical significance of snoRNAs and provide a promising biomarker for predicting and evaluating the clinical outcome of HNSCC patients.

## 2 MATERIALS AND METHODS

### 2.1 Data acquisition and preprocessing

We use the public database UCSC Xena (https://xenabrowser.net/datapages/) to download the head and neck squamous cell carcinoma (HNSCC) protein‐coding genes (PCGs) expression data in the form of fragments per kilobase of transcript per million fragments mapped (FPKM). After being transformed into log2 (FPKM + 1), the PCGs with average expression value >1 were retained. For the snoRNA expression profile of the TCGA HNSCC cohort, we downloaded the gene annotations (hg19) of 403 and 1,457 snoRNA genes from UCSC Genome Browser and GENCODE, respectively, and merged them as 1,524 unique snoRNA genes. HNSCC miRNA‐seq BAM files were downloaded from the Cancer Genomics Hub (CGHub; https://cghub.ucsc.edu). We kept the high‐quality samples with ≥50% QC‐passed reads and ≥80% reads mapped to human genome and the latest bam files if duplicated. We then mapped the reads to snoRNA genes and quantified the expression of snoRNAs as reads per kilobase per million (RPKM). snoRNAs with an average RPKM > 1 across samples in HNSCC were defined as detectable snoRNAs (SNORic; http://bioinfo.life.hust.edu.cn/SNORic; Gong et al., 2017). The expression data of snoRNA were then log2 (RPKM + 1) transformed for corresponding analysis. The corresponding clinical follow‐up information of HNSCC is also coming from UCSC Xena and the samples with survival time less than 30 days were removed.

### 2.2 Construction of the prognostic model

Five hundred and ten HNSCC patients were involved in the construction of the predictive model after samples without clinical information were excluded, and they were randomly divided into training and testing sets (7:3). Survival related snoRNAs were identified using the HNSCC patients accompanied by the snoRNA expression profile as well as their clinicopathological features. Next, the snoRNAs with a *p* < .05 were screened out as candidate snoRNAs which were passed on for LASSO regression. LASSO is a popular method for regression analysis with high‐dimensional features (Sauerbrei, Royston, & Binder, 2007), which has been widely used in the Cox proportional hazard regression model for survival analysis with high‐dimensional data (Tibshirani, 1997). LASSO Cox regression analysis was performed to select the most powerful prognostic markers from the candidate snoRNAs identified from the training set, which was then used for the multivariate Cox regression analysis to identify the possibilities of snoRNAs as independent prognostic markers from the most powerful snoRNAs. Meanwhile, a risk score model was also constructed from this step. The patients were divided into two groups, according to the median score. A Kaplan–Meier survival curve was plotted to analyze the difference between the two groups, while receiver operating characteristic (ROC) analysis was used to evaluate the efficiency of the predictive model, and AUC values were calculated to validate the performance of the prognostic predictors. To combine some basic clinicopathological information with the risk score model, a nomogram was plotted to predict the 1‐, 3‐, and 5‐year survival probabilities of HNSCC patients. All of these processes were performed by R software (version 3.5.1).

### 2.3 Correlation analysis of the five snoRNAs and annotation of their function

To determine the coexpression relationships between the five‐snoRNAs and PCGs, the Pearson's correlation coefficients were calculated, and the PCGs positively or negatively correlated with the five snoRNAs were considered as snoRNA‐related PCGs(we chose the top 300 correlated PCGs with adjusting *p* < .05 for each snoRNA) which were used to make functional annotation prediction for snoRNA(Fan & Liu, 2016; F. Liu, Xing, Zhang, & Zhang, 2019; R. Liu et al., 2017). To get further insight into the function of top 300 snoRNA‐related PCGs for each snoRNA, the Database for Annotation, Visualization, and Integrated Discovery (DAVID, https://david.ncifcrf.gov/) was employed to perform the gene ontology enrichment analysis (Huang da, Sherman, & Lempicki, 2009). The gene lists of top 300 PCGs were uploaded, then analysis was run with default parameters and we got the results of the biological process (BP) and the Kyoto Encyclopedia of Genes and Genomes (KEGG) pathway. The annotation term with *p* < .05 is regarded as of statistical significance and the results were presented by dotplot (ggplot2 package in R) like our previous studies did (Xing, Zhang, Feng et al., 2019; Zhang, Feng, Du et al., 2018; Zhang, Feng, Li, Guo, & Li, 2018).

### 2.4 Gene set enrichment analysis analysis

The mRNA expression data from HNSCC in this study was obtained from the public database TCGA‐GDC (https://portal.gdc.cancer.gov/), and was downloaded as raw count. The expression data was preprocessed according to the following procedure. First, we keep the genes with count larger than 1 in at least 50% of the samples. Next, the expression value was normalized using the R package edgeR, which was then transformed into log2 (expression value +1), and genes with the average value >1 were kept for gene set enrichment analysis (GSEA) analysis. GSEA software (Version:3.0; http://software.broadinstitute.org/gsea/index.jsp) and R package clusterProfiler(Yu, Wang, Han, & He, 2012) were employed to perform GSEA analysis between high and low‐risk groups to determine how the hallmark phenotypes and pathways differ between them. According to the five‐snoRNA signature, patients were ranked in descending order by risk score. The first 25% and last 25% of the patients were used to perform GSEA. Two annotated gene set files (h.all.v6.2.entrez.gmt and c2.cp.kegg.v6.2.entrez.gmt) were selected as the reference gene set to characterize the differences between the two groups according to the mRNA expression profile.

## 3 RESULTS

### 3.1 Clinical characteristics of the patients

**Figure 1:**
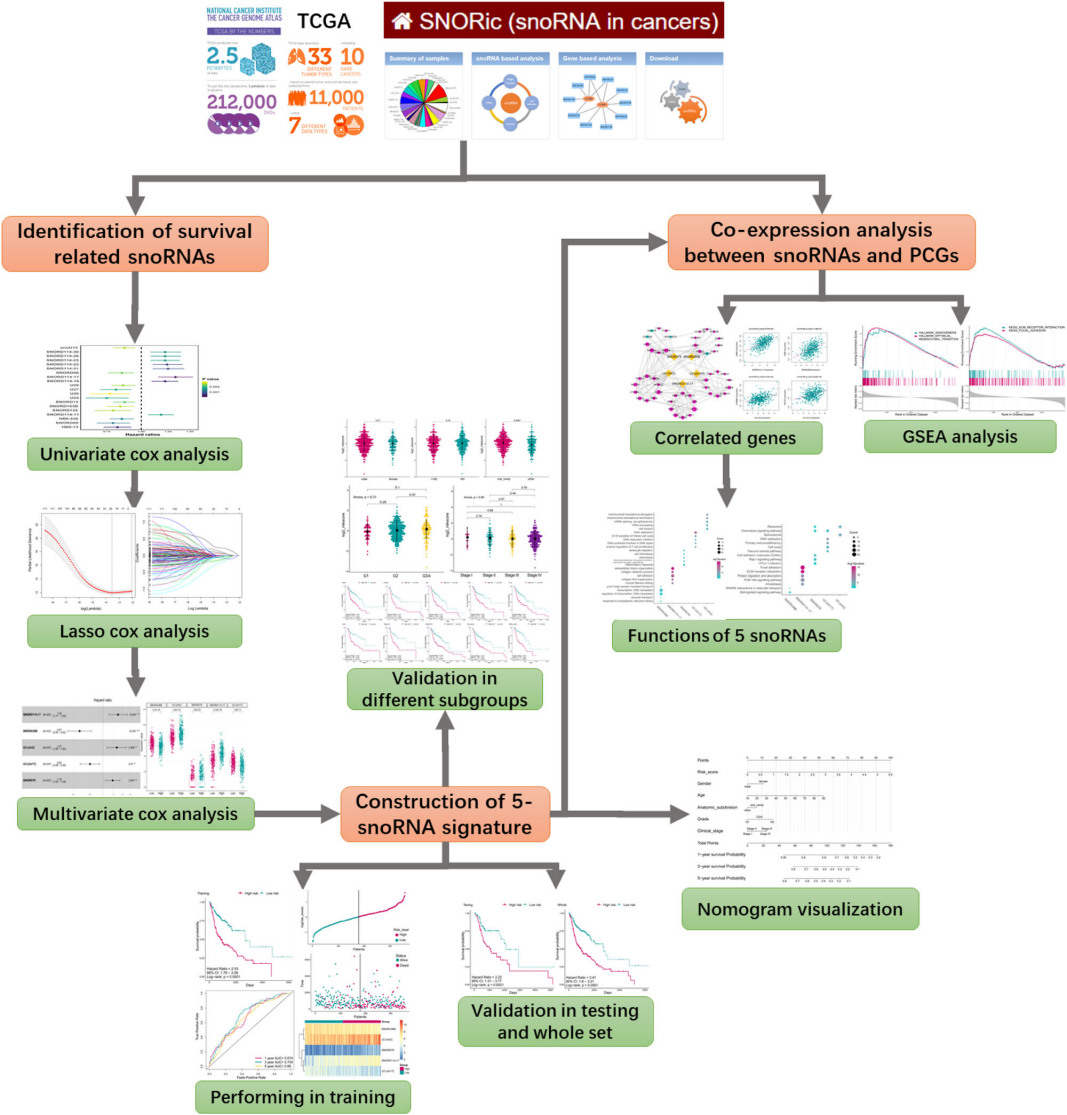
Flowchart of this study. PCG, protein coding genes; TGCA, The Cancer Genome Atlas; snoRNA, small nucleolar RNA

A total of 567 samples from the TCGA HNSC cohort were downloaded from the TCGA database, from which normal samples, samples without clinical follow‐up, as well as samples with overall survival time less than 30 days were excluded, thus 510 HNSCC samples were obtained for this study (Table 1). Five hundred and ten samples were further randomly divided into a training set and testing set at the ratio of 7:3 (357 and 153). The study workflow is demonstrated in Figure 1.

**Table 1:** Clinical characteristics of patients in head and neck squamous cell carcinoma cohort in this study

	Alive (*n* = 297)	Dead (*n* = 213)	Total (*n* = 510)
Gender			
Female	67	71	138
Male	230	142	372
Age			
Mean (SD)	59.55 (11.1)	62.77 (12.7)	60.9 (11.9)
Median [MIN, MAX]	60 [19, 85]	63 [24, 90]	61 [19,90]
Anatomic sub			
Oral cavity	184	149	333
Other	113	64	177
Clinical stage			
Stage I	14	7	21
Stage II	54	40	94
Stage III	60	44	104
Stage IVA	151	111	262
Stage IVB	5	4	9
Stage IVC	2	5	7
NA	11	2	13
Grade			
G1	39	23	62
G2	170	127	297
G3	68	55	123
G4	7	0	7
GX	10	7	17
NA	3	1	4

### 3.2 Prognostic values of snoRNAs

**Figure 2:**
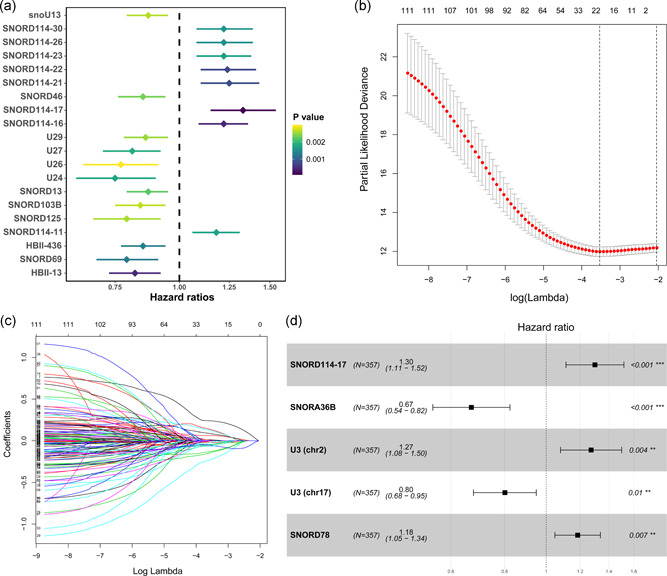
(a) The forestplot demonstrated the top 20 (20/113) significant survival‐related small nucleolar RNA (snoRNAs) using univariate cox analysis. (b) The tuning parameter (lambda) selection in the least absolute shrinkage and selection operator (LASSO) model used 10‐fold cross‐validation via minimum criteria. Dotted vertical lines were drawn at the optimal values by using the minimum criteria and the 1 standard error of the minimum criteria (the 1‐SE criteria). A lambda value of 0.029 was chosen (lambda.min) according to 10‐fold cross‐validation. (c) LASSO coefficient profiles of the 113‐survival related snoRNA. (d) Forestplot exhibited the results of multivariate cox analysis. Five significant snoRNAs in multivariate cox analysis were screened out (*p* < .05) as candidates for the risk model construction

Using univariate Cox proportional hazards regression analysis, the survival‐related snoRNAs were screened from training set, from which 113 snoRNAs were identified using *p* < .05 as the cut‐off. The top 20 significant survival‐related snoRNAs were demonstrated by the Forest plot (Figure 2a).

### 3.3 Construction of the prognostic snoRNA signature for HNSCC

A predictive model was constructed from 113 survival‐related snoRNAs using LASSO regression, and 19 snoRNAs with nonzero coefficients were selected with the minimum criteria (Figure 2b,c). These 19 snoRNAs were then passed on for multivariate Cox proportional hazards regression analysis, and snoRNAs with *p* < .05 were used for the predictive model construction. A five‐snoRNA (SNORD114‐17: ENSG00000201569, SNORA36B: ENSG00000222370, SNORD78: ENSG00000212378, U3: ENSG00000212182, and U3: ENSG00000212195)‐based risk model (Figure 2d) was eventually obtained, and a risk score formula was established according to their expression levels and coefficients. The five‐snoRNA risk score of each patient was calculated, and the patients were stratified into high‐ and low‐risk groups according to the median risk score.

### 3.4 Predictive value of the five‐pseudogene signature

**Figure 3:**
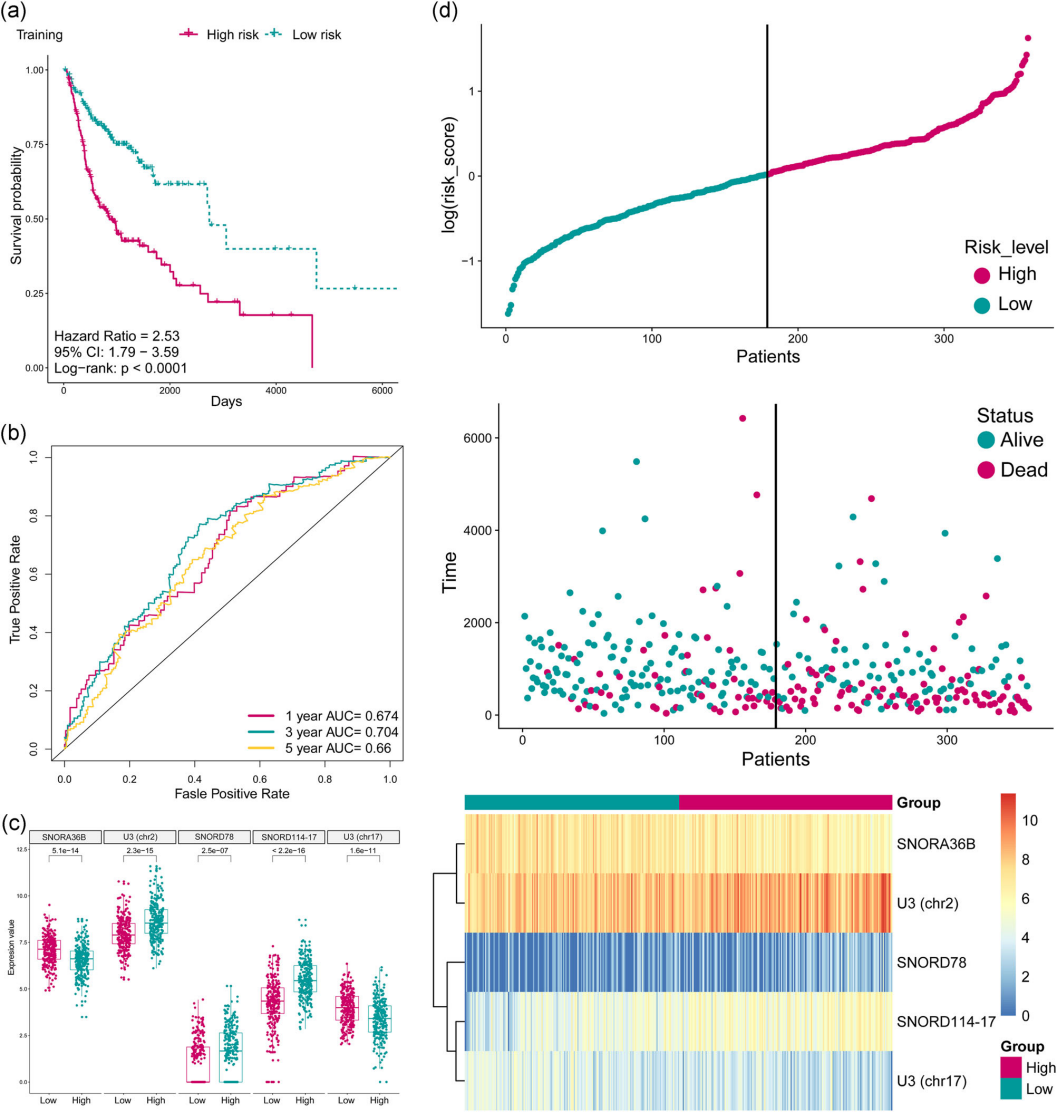
(a) The Kaplan–Meier plot of the overall survival (OS) for high‐risk and low‐risk patient cohorts divided by the five‐small nucleolar RNA (snoRNA) signature in the training data set (*N* = 357). The OS differences were determined by the two‐sided log‐rank test. (b) Receiver operating characteristic analysis for the five‐snoRNA signature in predicting the patients of 1, 3, and 5 years OS in the training set. (c) Boxplot displayed the expression status of the five snoRNAs between high risk and low‐risk group which was divided by the median risk score. (d) The distribution of the risk score, patients’ survival status as well as snoRNA expression signature in the training set. A shorter survival time, more dead events and the expression value of five snoRNAs ascended or decreased with the elevation of the risk score. AUC, areas under the ROC curve

**Figure 4:**
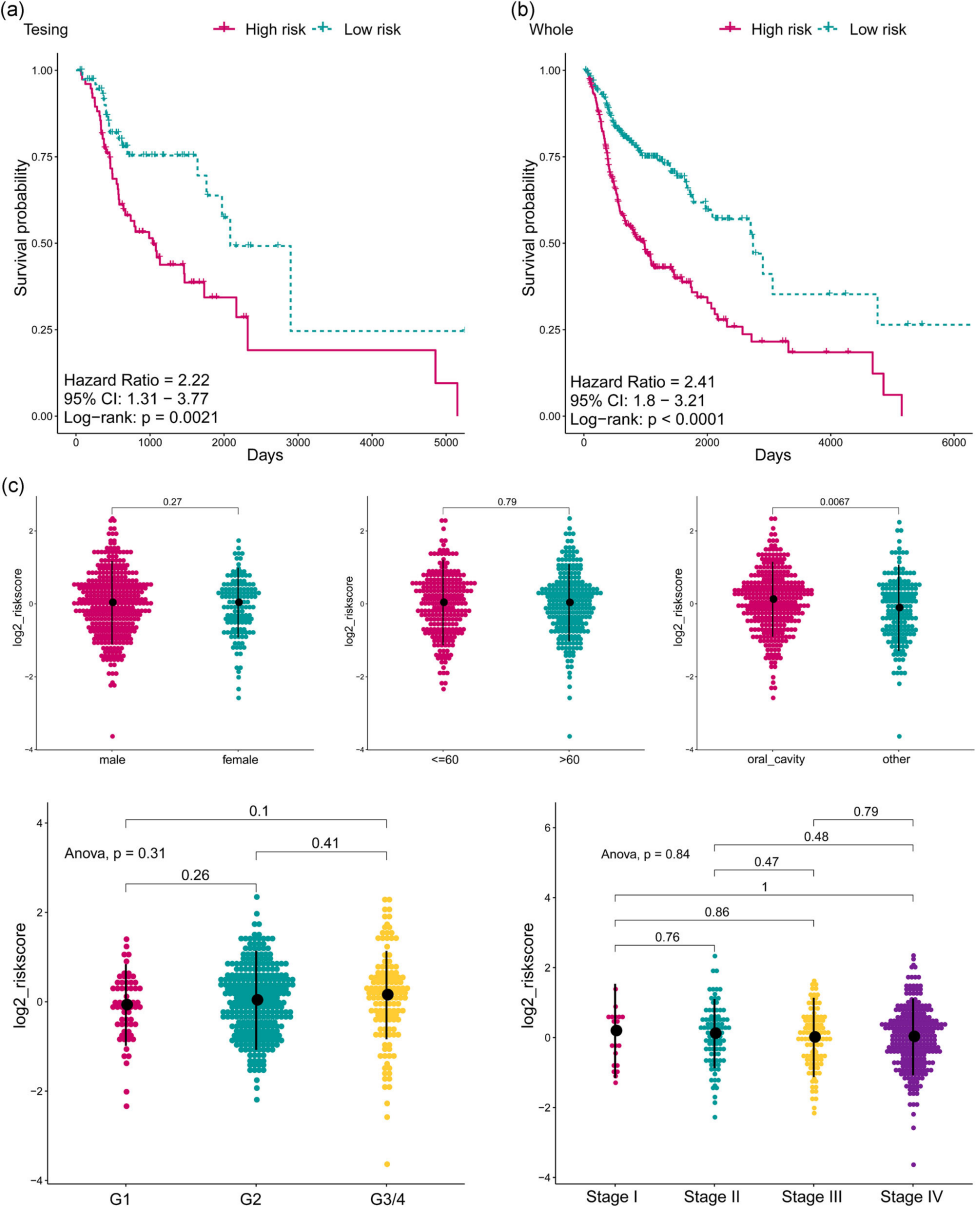
(a) The Kaplan–Meier plot of the overall survival (OS) for high‐risk and low‐risk patient cohorts divided by the five‐small nucleolar RNA (snoRNA) signature in the testing set (*N* = 153). The OS differences were determined by the two‐sided log‐rank test. (b) The Kaplan–Meier plot of the OS for high‐risk and low‐risk patient cohorts divided by the five‐snoRNA signature in the whole set (*N* = 510). The OS differences were determined by the two‐sided log‐rank test. (c) The dotplot exhibited the distribution of risk score based on the five‐snoRNA signature in different subgroups including genders, ages, anatomic subdivisions, grade and clinical stages. The risk score is not different in between gender, age, grade, and stage groups (*p* > .05). However, significant differences were observed between anatomic subdivisions (*p* < .05). ANOVA, analysis of variance

To reveal the potential prognostic value of the five‐snoRNA signature, Kaplan–Meier survival analysis was performed on the training set, and patients in the high‐risk group had a significantly worse prognosis (*p* < .0001; Figure 3a). Besides, ROC analysis was performed to evaluate the accuracy of this signature in predicting the 1‐, 3‐, and 5‐year survival (Figure 3b), the respective areas under the ROC curve (AUCs) were 0.674, 0.704, and 0.66, indicating a high sensitivity and specificity of this signature. SNORD114‐17, SNORD78, and U3 (chr2) were upregulated in the high‐risk group, which means that it is a risk factor for HNSCC, and the other three were downregulated in the high‐risk group, indicating that they act as protective factors (Figure 3c). In addition, a higher risk score was related to shorter overall survival, more death events, and higher expression levels of the five signature snoRNAs (Figure 3d). To further evaluate the predictive performance of the snoRNA signature, it was applied to the testing set and the whole set, and similar results were obtained (Figure 4a,b). In conclusion, this five‐snoRNA signature‐based risk model can well distinguish high‐risk patients from low‐risk patients with HNSCC, indicating its prognostic significance for HNSCC.

### 3.5 Predictive performance of the five‐snoRNA signature in different subgroups

**Figure 5:**
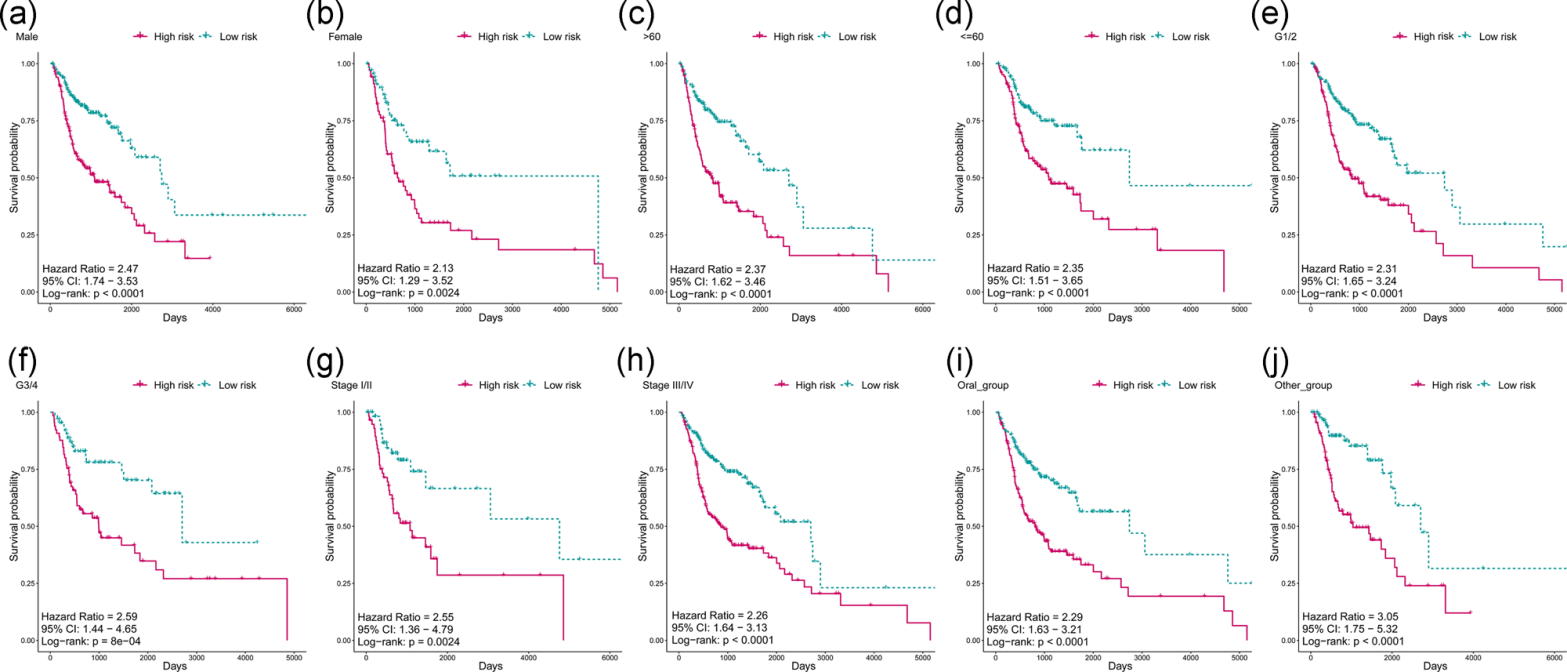
(a–j) Kaplan–Meier analyses of patients with head and neck squamous cell carcinoma in different subgroup cohorts, patients were grouped based on their gender, age, subdivision, grade, and stage. Kaplan–Meier analysis with a two‐sided log‐rank test was performed to estimate the differences in overall survival between the low‐risk and high‐risk patients

To identify other likely contributors such as sex, age, clinical stage, histological grade, and other clinical features on patient survival, the patients were also grouped according the above variables and then we applied the five‐snoRNA signature to the different subgroups. Since differences between different subgroups might influence the performance of the prognostic biomarkers. When it comes to sex subgroups, there were 372 males and 138 females in the HNSCC cohort, which did not differ significantly in terms of the risk score distribution (Figure 4c). Besides, the high‐risk patients also had significantly shorter OS in both the male and female groups (Figure 5a,b), which means that the five‐snoRNA signature was independent of sex. There was also no significant difference in the risk score distribution of younger (≤60 years, *N* = 251) and older (>60 years, *N* = 259) patients (Figure 4c). Similarly, in between this subgroup, the low‐risk patients had significantly longer overall survival (Figure 5c,d), indicating the independence of the five‐snoRNA signature in age subgroups. As for histological grade subgroups, the patients were divided into grade 1/2 and grade 3/4 groups. And significantly shorter OS was observed in grade 3/4 subgroup compared to the grade 1 and 2 patients. Risk score distribution also did not differ significantly among these groups (Figure 4c). Regardless of tumor grade, overall survival was significantly different between the high‐ and low‐risk groups (Figure 5e,f), indicating that the five‐snoRNA signature was an independent predictor of histological grade. According to the clinical stage, patients were stratified into the clinical Stage I/II and Stage III/IV groups, and similar risk scores were also observed (Figure 4c). Besides, the patients in the high‐risk group had a significantly poorer prognosis than the low‐risk group in both the Stage III/IV and Stage I/II subgroups (Figure 5g,h), indicating good performance of the five‐snoRNA signature in stage subgroups. Based on anatomic neoplasm subdivision, patients were divided into the oral cavity and other groups. However, significant differences were observed between anatomic subdivisions (*p* < .05; Figure 4c). Likewise, the OS was significantly different between the high‐ and low‐risk groups regardless of subdivision (Figure 5i,j), indicating its independence. In a nutshell, the five‐snoRNA signature can be applied to HNSCC patient subgroups stratified based on clinical‐pathological features and is an independent prognostic signature for HNSCC.

### 3.6 Nomogram and calibration

**Figure 6:**
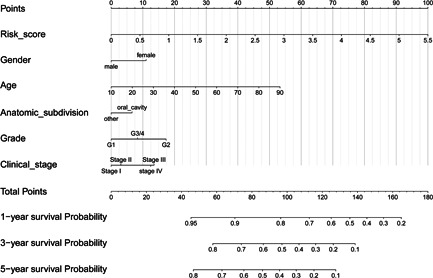
Nomograms combining five‐small nucleolar RNA signature and clinicopathological features to predict 1‐, 3‐, and 5‐years survival probability of patients with head and neck squamous carcinoma

A multivariate Cox proportional hazard regression model was constructed to combine some clinicopathological features (age, anatomic subset, sex, clinical stage, and histological grade) with the five‐snoRNA signature for prediction of patients prognosis of 1‐, 3‐, and 5‐year survival probability. A nomogram was plotted to visualize this model with an assigned score for each term (Figure 6). It can also be concluded that the five‐snoRNA signature is independent of other clinicopathological features in predicting the patient's survival.

### 3.7 Functional analysis of the predictive snoRNAs

**Figure 7:**
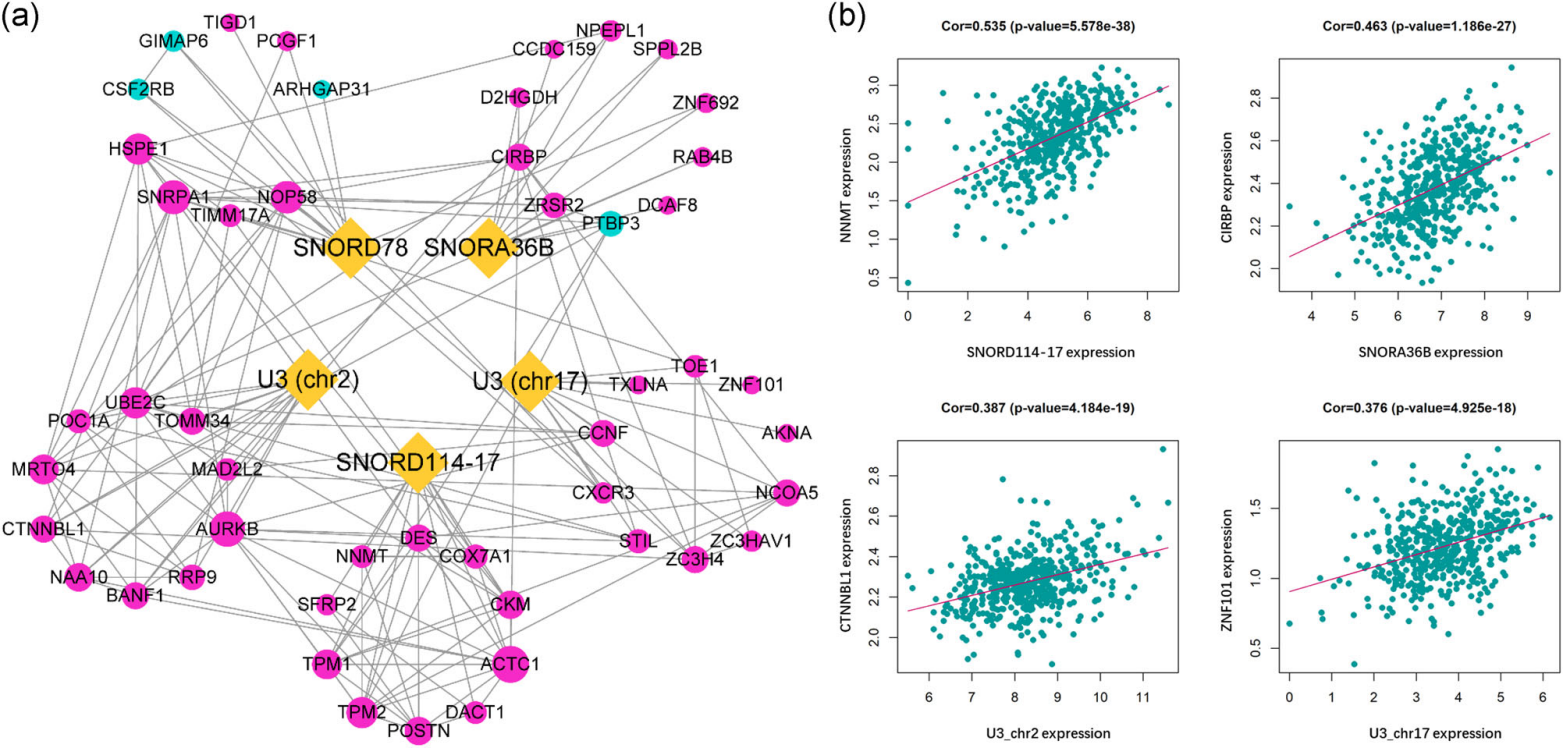
(a) The network of five snoRNAs and their top 10 co‐expressed PCGs. The correlation analysis between snoRNAs and PCGs were performed by Pearson correlation analysis. The interaction among PCGs was generated by the STRING database. Yellow dots represent snoRNAs, red dots present positive correlated genes while blue dots represent negative corelated PCGs. The dot size represents a degree. (b) The representative of corelated PCGs. The dotplots demonstrated the most correlated genes of each snoRNAs. HSNCC, head and neck squamous carcinoma; PCG, protein coding genes, snoRNA, small nucleolar RNAs

**Figure 8:**
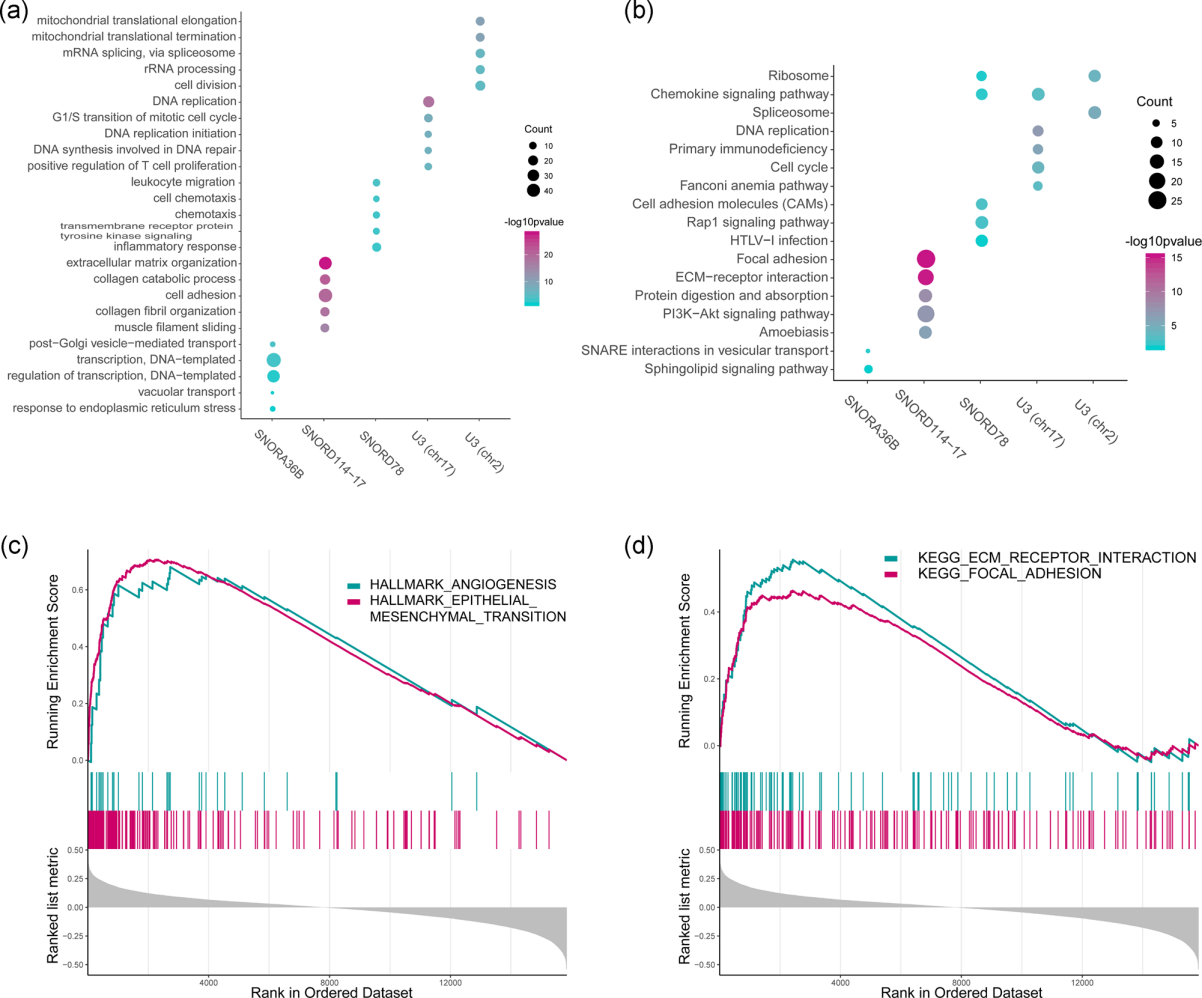
(a) The gene ontology enrichment analysis for the five snoRNAs correlated PCGs were carried out using DAVID to reveal the potential function of the five snoRNAs. The top five significant biological process terms for each snoRNA were shown. (b) The KEGG pathway enrichment analysis for the five snoRNAs correlated genes were carried out in DAVID to reveal the potential pathways in which the five snoRNAs are involved. The top five significant pathway terms for each snoRNA are shown. (c) GSEA enrichment analysis showed the significantly enriched HALLMARK terms associated with the risk score. (d) GSEA enrichment analysis showed the significantly enriched KEGG pathways terms associated with the risk score. DAVID, Database for Annotation, Visualization, and Integrated Discovery; GSEA, gene set enrichment analysis; KEGG, Kyoto Encyclopedia of Genes and Genomes; PCG, protein coding genes, snoRNA, small nucleolar RNAs

To identify the protein‐coding genes co‐expressed with the snoRNAs, correlation analysis was performed, using the top 300 co‐expressed PCGs, followed by Gene Ontology (GO) and Kyoto Encyclopedia of Genes and Genomes (KEGG) enrichment analysis, the function of the predictive snoRNAs were determined. A network was presented to demonstrate the relationship of the top 10 correlated PCGs of each snoRNA (Figure 7a). The PCGs with the highest correlation with each snoRNA were as follows: SNORD114‐17‐NNMT, SNORA36B‐CIRBP, U3(chr2)‐CTNNBL1, and U3(chr17)‐ZNF101 (Figure 7b). The functions of the snoRNAs as per GO biological processes (BP) and KEGG are shown in Figure 8a,b; SNORA36B is involved in DNA template regulation, SNORD114‐17 is involved in regulation of cell adhesion, invasion, and metastasis, U3 (chr2) is related to RNA editing, and U3 (chr17) plays a role in cell proliferation, and SNORD78 has multiple functions. In addition, SNORD114‐17 is involved in several well‐known cancer‐related pathways, such as PI3K‐AKT signaling and the ECM receptor, suggesting that SNORD114‐17 may be an important regulator of the malignant phenotype. Additionally, GSEA was performed to identify snoRNA‐related hallmarks enriched in the high‐risk group. Several cancer‐related processes, including EMT and angiogenesis, were significantly enriched in the high‐risk group (Figure 8c). The KEGG pathways associated with HNSCC, such as ECM receptor and focal adhesion, were significantly enriched (Figure 8d). In a word, the five snoRNAs are associated with tumor progression.

## 4 DISCUSSION

Patients with HNSCC frequently have a poor prognosis and a low survival rate. Despite great improvements in diagnostic and therapeutic methods, the survival rate of HNSCC is still low (Troiano et al., 2018). Prognostic biomarkers can provide information about the probable outcome of a cancer relative to disease progression, recurrence, or death (Ballman, 2015). This information could greatly aid in patient stratification, treatment management and monitoring disease status in clinical practice, for example, offering personalized therapeutic schedules to HNSCC patients who would benefit enormously (Nonaka & Wong, 2018). The prognostic prediction would be very useful for clinicians to aid in choosing the magnitude and type of therapeutic approach (surgery, chemotherapy, radiotherapy, or a combination of these) on the basis of the molecular profile of HNSCC (Troiano et al., 2018). In the past few years, multiple molecular biomarkers have been proven to predict the clinical prognosis in different kinds of cancers (Quan et al., 2018; Zhang, Feng, Li, Liu et al., 2018; Zhu et al., 2016). In addition, combining several biomarkers achieved higher sensitivity and specificity compared to individual markers (Guo et al., 2018; Zhao, Sun, Zeng, & Cui, 2018). Therefore, a predictive model based on several signatures for various molecules, such as mRNA (Zhang, Feng, Li, Li et al., 2018), miRNA (Wong et al., 2016), lncRNA (G. Liu et al., 2018), and methylation (Shen et al., 2017), was found in HNSCC.

We focused on snoRNAs and used similar methods to identify and prove that snoRNA could also serve as a prognostic signature. snoRNAs are a class of small (60–300 nt) noncoding RNAs implicated in the chemical modification of rRNA and are commonly known as housekeeping genes. They have been known to function as a guide for the posttranscriptional modification of rRNA, but in recent years, a new role in the regulation of other cellular pathways has emerged (Scott & Ono, 2011) including the regulation of oncogenesis in different cancers (Gong et al., 2017). Compared with the well‐characterized role of miRNAs in biomarkers for early detection, recurrence and prognostication prediction (Hayes, Peruzzi, & Lawler, 2014), little is known about the significance of snoRNAs. Furthermore, the association between snoRNA expression and their clinical impact as biomarkers for HNSCC has not been undertaken. Therefore, we performed a systematic analysis of the potential role of snoRNAs as prognostic predictors and provided the first evidence of survival‐related snoRNAs. We made several important discoveries during the course of this analysis. First, we identified 113 survival‐related snoRNAs using univariate Cox analysis, most of which are protective factors (83/113), which may play a role in tumor suppression. Second, we identified a five‐snoRNA signature and established a scoring system that was significantly associated with the OS of HNSCC patients. This signature helped stratify low‐ and high‐risk groups and predicted the OS of HNSCC patients with high sensitivity and specificity. The signature was first constructed in the training set and then validated in the testing set and whole set, suggesting that it was reliable. Two of the five snoRNAs, SNORA36B, and U3 (chr17), are protective factors, and the other three are risk factors. A previous study reported that SNORD78 greatly upregulated in non‐small cell lung cancer tissues and inhibition of SNORD78 suppressed the proliferation of NSCLC cells via inducing G0/G1 cell cycle arrest (D. Zheng et al., 2015), and Langhendries, Nicolas, Doumont, Goldman, & Lafontaine (2016) revealed in a mouse xenograft model that the tumorigenic potential of cancer cells was reduced in the case of U3 suppression. As for SNORD114‐17 and SNORA36B, there are no related reports in the cancer field. Third, to validate the universality in different patients and extend the signature to various subgroups, Kaplan–Meier survival analysis was performed in different subgroups. We found that the five‐snoRNA signature was independent of other potential predictors, including age, sex, anatomical subdivision, clinical stage and histological grade, and the performance of predicting survival was satisfactory. We visualized the snoRNA signature and the other clinical information by a nomogram to simplify the use of this signature in clinical practice. However, due to the incompletion of the clinical data, we can get, rare patients have gone through chemotherapy or radiation therapy before the samples were taken (most of patients history of neoadjuvant treatment are NO), and most of them have gone through chemotherapy or radiation therapy after the sample collection which means these samples are almost not influenced by chemotherapy or radiation therapy. So we can't analyze the influence of radiotherapy or chemotherapy in the expression level of snoRNA, which in turn may affect the results of the data analysis. Further studies with more complete clinical information should be carried out. Finally, to further understand the biological function and explore the underlying oncogenic mechanism of the five snoRNAs, co‐expression analysis, and GSEA were employed. The results showed that the five snoRNAs were involved in some well‐known cancer‐related pathways, such as the PI3K/AKT and ECM‐receptor. Some functions concerning DNA/RNA editing and cell proliferation were also presented.

The overall survival of patients with HNSCC can be multifactorial and cannot be solely determined by genomic and transcriptomic dysregulation, and risk factors included TNM staging, primary site, histological grade and treatment (Carrillo, Carrillo, Ramirez‐Ortega, Ochoa‐Carrillo, & Onate‐Ocana, 2016). Since most of the conventional therapies treating HNSCC can be very toxic, patient stratification using validated biomarkers can be very important to improve treatment outcomes and reduce toxicity and cost of HNSCC treatment (Alsahafi et al., 2019). Nowadays, with the rapid development of high throughput RNA sequencing, the transcriptomic alterations behind HNSCC have been gradually revealed, and a large number of molecular signatures have been developed to enhance the stratification of HNSCC. In our study, we aim to construct a signature based on snoRNA alterations for the classification of HNSCC patients in overall survival, which could probably help with the treatment and follow‐up management of HNSCC patients. For example, imagine two HNSCC patients with the same age, sex, stage, and grade, who might be classified into the same category which indicates a certain prognosis. However, the prognostic outcome of them might not be the same, and quantifying their prognosis can be tricky. In our study, we integrated the risk score and other clinical risk factors to provide the clinicians a quantitative approach to predict the prognosis of HNSCC patients, a better way to both combine the snoRNA profile and clinicopathological for overall survival prediction. We believe the biomolecular signature will make great assistance for doctors in clinical practice accompanied by the development of high‐throughput sequencing technology.

There are some limitations and shortcomings in this study that cannot be ignored. First, this study mainly focused on data mining and data analysis, which are based on methodology, and the results were not validated using experiments. For example, the snoRNA co‐expressed PCGs as functional annotation prediction of snoRNA is based on previous studies and is theoretically feasible. Experiments are needed to confirm the predictive results and identified the precise function and related pathways that the five snoRNAs involved in. Further validation experiments are required to verify the findings of this study. Second, the datasets we were able to obtain were limited, as we could obtain only one The Cancer Genome Atlas (TCGA) HNSCC data set that contained both HNSCC patient miRNA‐seq data and clinical follow‐up information. If there were other datasets that met with our requirements, these could have been used to further validate our results. Additional datasets should be included to obtain a better result. Third, when constructing a snoRNA signature for prognosis, one must take it into consideration of the application of such a model. Since different methods of detecting snoRNAs might lead to different results, the procedure of detection, quantification, and determination of transcriptomic activity of snoRNAs must be standardized (Guglas et al., 2017). Last, miRNA‐seq is not designed for a full snoRNA repertoire, as miRNA‐seq reads are too short (15–30 bp) to distinguish snoRNA from snoRNA fragments (Krishnan et al., 2016), which may have different biological functions. However, this method has been applied in several studies (Gao et al., 2015; Krishnan et al., 2016; L. L. Zheng et al., 2016) and is probably the most appropriate way to quantify snoRNA expression profiles from TCGA omics data. Thus, validating full‐length snoRNAs from miRNA‐seq data for further investigation is necessary (Gong et al., 2017). Therefore, the five newly found prognosis‐related snoRNAs deserve more attention, and the next step for our research is to validate our results using experiments. We hope that these results could give other researchers inspiration for further exploration.

Taken together, we identified a novel five‐snoRNA signature for HNSCC that is a promising independent survival predictor and serves as an important biomarker for guiding the clinical treatment of HNSCC patients to improve patient management. In addition, our findings provide new insights into the molecular mechanisms underlying HNSCC and present a promising new prognostic marker. Therefore, our findings in the signature have very promising clinical significance.

## 5 CONCLUSIONS

In conclusion, this study highlighted the prognostic value of snoRNAs and explored their underlying functions. Some prognosis‐related snoRNAs have been revealed, and the survival of HNSCC patients could be predicted by a risk model based on these snoRNAs, which could serve as prognostic markers in clinical practice. These results may provide new potential prognostic and therapeutic implications for HNSCC patient management.

## CONFLICT OF INTERESTS

The authors declare that there are no conflict of interests.

## AUTHOR CONTRIBUTIONS

Conceptualization, L. X., X. Z., and D. T.; Data curation, L. X. and X. Z.; Formal analysis, L. X. and X. Z.; Funding acquisition, D. T.; Methodology, Xiaoqian Zhang; Project administration, D. T.; Software, X. Z.; Supervision, D. T.; Validation, X. Z.; Visualization, L. X. and X. Z.; Writing–original draft, L. X. and X. Z.; Writing–review & editing, D. T. L. X. and X. Z. contributed equally to this work and should be consider as co‐first authors.

## Supporting information

Supporting information

## Data Availability

The data that support the findings of this study are available in [UCSC Xena] at [https://xenabrowser.net/datapages/] and [SNORic] at [http://bioinfo.life.hust.edu.cn/SNORic].
