# Supplementary material for: Expression scoring of a small‐nucleolar‐RNA signature identified by machine learning serves as a prognostic predictor for head and neck cancer
Source: J Cell Physiol. 2020 Jan 14;235(11):8071–84. doi: 10.1002/jcp.29462 (PMC7540035; doi:10.1002/jcp.29462)
Supplement: Supplementary file 1 — Supporting information [file JCP-235-8071-s001.docx]

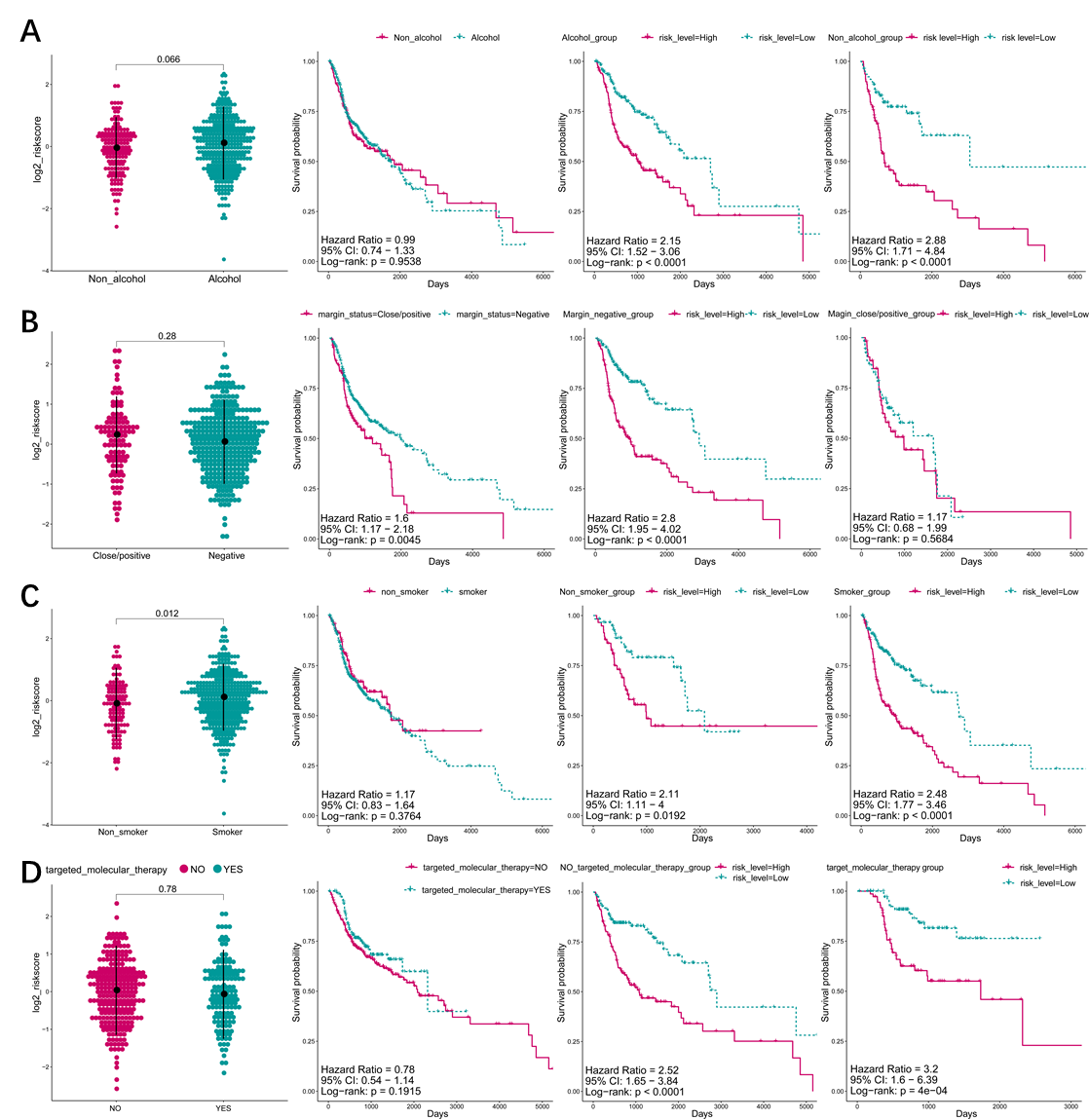


**Figure S1:** (A-D) Dotplots and Kaplan–Meier analyses of patients with HNSCC in different subgroup cohorts, grouping based on their alcohol status, margin status, smoking status and target molecular therapy status. Kaplan–Meier analysis with two-sided log-rank test was performed to estimate the differences in OS between the low-risk and high-risk patients.
